# Robust Kernel-Based Tracking with Multiple Subtemplates in Vision Guidance System

**DOI:** 10.3390/s120201990

**Published:** 2012-02-10

**Authors:** Yuzhuang Yan, Xinsheng Huang, Wanying Xu, Lurong Shen

**Affiliations:** College of Mechatronics Engineering and Automation, National University of Defense Technology, Changsha 410073, China; E-Mails: huangxinsheng@163.com (X.H.); wanying xu@nudt.edu.cn (W.X.); shenlurong09@gmail.com (L.S.)

**Keywords:** tracking, mean shift, subtemplate, voting, vision guidance

## Abstract

The mean shift algorithm has achieved considerable success in target tracking due to its simplicity and robustness. However, the lack of spatial information may result in its failure to get high tracking precision. This might be even worse when the target is scale variant and the sequences are gray-levels. This paper presents a novel multiple subtemplates based tracking algorithm for the terminal guidance application. By applying a separate tracker to each subtemplate, it can handle more complicated situations such as rotation, scaling, and partial coverage of the target. The innovations include: (1) an optimal subtemplates selection algorithm is designed, which ensures that the selected subtemplates maximally represent the information of the entire template while having the least mutual redundancy; (2) based on the serial tracking results and the spatial constraint prior to those subtemplates, a Gaussian weighted voting method is proposed to locate the target center; (3) the optimal scale factor is determined by maximizing the voting results among the scale searching layers, which avoids the complicated threshold setting problem. Experiments on some videos with static scenes show that the proposed method greatly improves the tracking accuracy compared to the original mean shift algorithm.

## 1. Introduction

Target tracking is one of the most important tasks in computer vision. It serves as the foundation for numerous higher-level applications in many domains, including video surveillance, visual based navigation and precision guidance, *etc*. In this study, we focus on the tracking problem of vision-based terminal guidance system. After capturing the target from the first frame by the human in loop or some other automatic target recognition (ATR) system, a robust and accurate tracking program directly determines the final precision. However, as the imaging system of the terminal guidance is generally lack of color information, and the target presents complicated changes such as translation, rotation ([0, 2*π*]) and scale (*>*1) while the attitudes and position of the carriers alter successively, it is a very difficult task to achieve a robust tracking.

As introduced in [1], there exists many tracking algorithms, such as Lucas–Kanade [2], mean shift [3,4], template matching [5]. Among the various tracking algorithms, mean shift, also known as kernel based tracking, has attracted much attention in the computer vision community since 2000 [3,6–9].

The mean shift can be deemed as an optimal hill-climbing algorithm with adaptive step sizes. It finds the local maxima by a similarity measure between the color histograms or kernel density estimates of the model (template) and the target image. The most typically used similarity measure is the Bhattacharyya coefficient. Mean shift is relatively simple and computationally efficient, and is rotational invariant as long as the template is circular, and it can be easily extended to be scale adaptive [10], which is achieved by increasing or decreasing the size of the candidates (corresponding to the bandwidth of kernel function). However, the original mean shift tracking suffers from an obvious weakness due to the lack of spatial color information of the target, which makes it often trapped in a wrong position [11], especially in the gray-level cases. Ning *et al*. introduced the moment features of weight image (*w_i_*) to estimate the scale and orientation, named by SOAMST (scale and orientation adaptive mean shift tracking). The SOAMST performs robust when the target region are obviously different from its background in color, however, as the tracking features are not changed, its localization ability is not improved.

Therefore, various multi-part models have been proposed to enforce the particular spatial structure of the target. Maggio *et al*. propose a multi-part target representation based on the computation of seven semi-overlapping color histograms [12]; Calfield *et al*. combine the background exclusion constraints with multi-part appearance models [11]; Jia and Zhang propose a multiple kernel based target tracking method which divides the target into blocks and extracts kernel weighted histograms of oriented gradients for each block [13]. These methods significantly improve the spatial description ability of the original mean shift and provide higher tracking precision, however, none of them can cope with the rotation changes, which is critical to our application. Xu *et al*. propose a spatial color histogram, which divide the target region into some circular ring-like regions, to incorporate spatial information. This method makes the surface of Bhattacharyya coefficients more convex, and improve the tracking accuracy [14]. Yilmaz proposes an asymmetric kernel based tracking method to solve the anisotropic target problem, which is suitable for scale and rotation changes. However, its computation is costly [15].

It is well known that feature would be more distinguished by increasing its dimension. In this paper, we will introduce a new multi-part tracking method based on the mean shift algorithm. This method uses multiple trackers to improve the tracking precision, and each tracker works based on a unique subtemplate, which is cropped from the target template. First, we optimally select a number of circular subtemplates, and enforce them to best represent the original target template. Meantime, we take their spatial distances to the target center as a priori knowledge. Then each subtemplate is respectively tracked by the original mean shift algorithm, and a number of tracking results can be obtained. Next, a voting strategy is designed to determine the real target center. At each tracking position, we define each voter by drawing a Gaussian weighted band-ring like surface with the recorded distance prior as its radius, and by overlapping those surfaces and finding its maximum, we can locate the target center. Each tracker is inherently rotational invariant, so their combination is also rotational invariant. Finally, to cope with the scale changes, a scale searching is conducted by changing the statistical radius of the candidate target (new scale layer) and repeating the above steps (tracking and voting), and the best scale factor can be obtained form the maximum voting result of those layers.

The remainder of this paper is organized as follows. Section 2 introduces the original mean shift tracking theory. The proposed method, including the optimal subtemplates, target center voting and the scale searching, is introduced in Section 3. Experimental results are presented in Section 4. Finally, in Section 5 we draw the conclusions.

## 2. Original Mean Shift Tracking

Consider two images, one designated as the model image which includes the targets for tracking and the other the target image in which we need to find the targets. The sample points in the model image are denoted by *I_x_* = {*x_i_*}*_i_*_=1_*,...,N*, where *x_i_* is the 2D coordinates. Given the kernel function *g*(*x*), the probability density function (PDF) of the target in the model image, which is denoted by **q** = {*q_u_*}_*u*=1...*m*_, can be estimated using kernel density estimation. Here, *m* is the feature dimension (bin’s number). For each bin *u*,

(1) $$q_{u}(x_{0})=C_{h0}\sum_{i=1}^{N}g\left({\left\|\frac{x_{i}-x_{0}}{h_{0}}\right\|}^{2}\right)\;\;\delta \;[b\;(x_{i})-u]$$

where *x*_0_ is the target center; *C*_*h*0_ is a normalization constant, which satisfies $\sum_{u=1}^{m}q_{u}=1$; *h*_0_ is the bandwidth of *g*, which corresponds to the size of the model image; *δ* is the Kronecker function; *b* returns the feature value at *x_i_*.

Similarly, given the sample points of the candidate region in the target image, which is denoted by *I_y_* = {*y_i_*}_*i*=1,...,*M*_, its PDF **p** = {*p_u_*}_*u*=1...*m*_ is given by

(2) $$p_{u}(y)=C_{h1}\sum_{i=1}^{M}g\left({\left\|\frac{y_{i}-y}{h_{1}}\right\|}^{2}\right)\;\;\delta \;[b\;(y_{i})-u]$$

where *y* is the center point of the candidate region; *h*_1_ is the new bandwidth corresponding to *M*, and *h*_1_ = *h*_0_ when the target size is constant in the tracking.

The similarity of the two PDFs are measured by Bhattacharyya distance. Based on the Epanechnikov kernel function, Comaniciu *et al*. derived the mean shift iterative procedure as [6]

(3) $$\hat{y}=\frac{\sum_{i=1}^{M}y_{i}w_{i}}{\sum_{i=1}^{M}w_{i}}$$

where

(4) $$w_{i}=\sum_{u=1}^{m}\sqrt{\frac{q_{u}}{p_{u}(y_{0})}}\delta \;[b\;(y_{i})-u]$$

is the weight for the *i*th pixel. The tracker is considered to be convergent if the distance between two successive iterations is smaller than a particular threshold.

## 3. Proposed Method

The proposed method mainly includes three steps: (1) select *N* circular subtemplates according to a certain criteria; (2) conduct tracking with those subtemplates, and determine the target center by a novel voting strategy; (3) obtain the scale factor by searching with different patch size for each candidate.

### 3.1. Optimal Subtemplates Selection and Tracking

**Figure 1.**
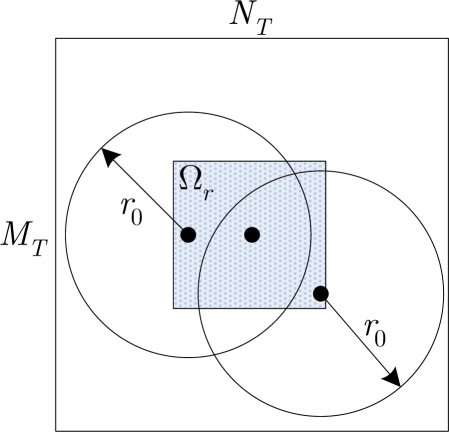
The subtemplate distribution.

Given an *M_T_ × N_T_* template *T*, from hundreds of candidate points (supposed to be *M*), we are going to find the best *N* subtemplates with the same size. Let *r*_0_ be the radius of subtemplate, and ${\left\{x_{0}^{n}\right\}}_{n=1,\ldots ,N}$ be their corresponding centers. Figure 1 shows a sketch map of the subtemplate distribution, where all the *M* potential candidates are restricted within the inner region Ω*_r_*, whose boundaries are *r*_0_ away from the template boundaries (marked in shadow).

The selection relies on the following three rules: ① scattered spatial distribution: the selected subtemplates should cover the template region as much as possible; ② high identifiability: to improve the tracking precision, each subtemplate should have a high identifiability, which can be evaluated by the self-similarities to its neighbors; ③ minimum redundancy: to best represent the potential properties of the target and to minimize their mutual redundancies, those subtemplates should maximize their dissimilarities to each other.

Based on these rules, we design the selection process as described in Algorithm 1. First, we calculate all the candidates’ kernel histogram by Equation (1), which is denoted by {**q***_i_*}, *i* ∈ Ω*_r_*. Then the first subtemplate is selected by maximizing the self-dissimilarity based on rule ②. Here, the dissimilarity is evaluated by the *L*_2_ norm. The rest of the subtemplates are serially selected according to rules ① and ③. Their processes correspond to the iteration steps (lines 5–10) in Algorithm 1, in which lines 6–7 correspond to rule ③ and lines 8–9 correspond to rule ①, respectively.

**Algorithm 1:**
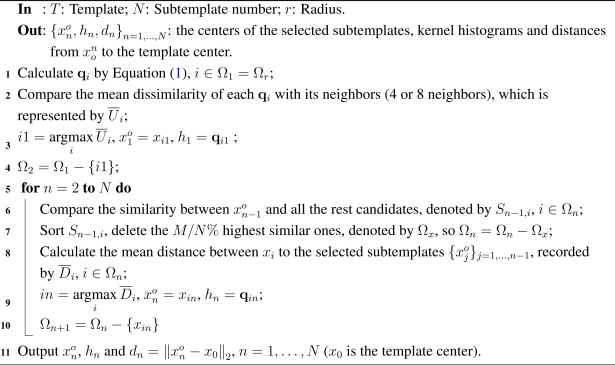
Subtemplates selection.

**Figure 2.**
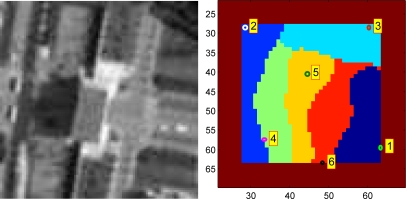
Template (left) and the centers of the selected subtemplates (right).

To better understand this algorithm, an example is presented, as shown in Figure 2, the template size is 90×90 (left), *r*_0_ = 27 and *N* = 6. In the right figure, all the subtemplate centers and their corresponding serial number during the iteration are marked out. Meanwhile, the (*M/N*)% highest similar candidates to each selected subtemplate are marked by a unique color. It can be seen that each center point holds a separate subregion, and all of them scatter in the full template region and in the meantime they are located far away from each other. In this example, the centers of the 6 selected subtemplate are: (59,63), (28,28), (28,60), (57,33), (40,44), (63,48), and their distances from the target center are: (21.4, 25.4, 22.8, 17.0, 6.3, 17.1).

With each subtemplate, we can conduct the mean shift tracking according to the equations discussed in Section 2, and the *N* tracking results, ${\left\{x_{n}^{*}\right\}}_{1,\ldots ,N}$, can then be obtained.

In the next section, we are going to introduce the voting based target center determining method. For facilitating the analysis, we suppose the target is in fixed-scale mode. In other words, the kernel bandwidths for the histogram statistic in both model and target images are constant, which equals *r*_0_. The variable-scale tracking problem will be discussed in Section 3.3.

### 3.2. Voting the Target Center

Our voting strategy is very simple and novel. In the last subsection, we have obtained the priori distances {*d_n_*}_*n*=1,...,*N*_ from each subtemplate to the target center, which can be treated as the geometric constraints of the subtemplates. Obviously, the *N* tracking results should also satisfy these constraints when the target is in fixed-scale mode. By this prior, suppose that we draw *N* circles centered at ${\left\{x_{n}^{*}\right\}}_{1,\ldots ,N}$, respectively, the point with highest cross times within those rings must be the target center. Ideally, the cross times should be 6. However, not all the tracking results are reliable. For achieving a more robust location, each voter is modeled by a Gaussian weighed function, with $x_{n}^{*}$ as its center and the prior distance *d_n_* as its radius.

(5) $$W_{n}(x)=\frac{1}{2\pi \sigma^{2}}\;\text{exp}\;\left(\frac{-{\left(D_{n}(x)-d_{n}\right)}^{2}}{2\sigma^{2}}\right)$$

where $D_{n}={\left\|x-x_{n}^{*}\right\|}_{2}$ is the distance between *x* and $x_{n}^{*}$; *σ* is the bandwidth of the Gaussian function, which is used for estimating the inherent error of the kernel histogram based tracking methods. Obviously, the greater *σ* is, the higher error tolerance the voting will have. Generally, an error exceeding ±5 pixels is deemed as invalid tracking, we set *σ* = 4/3 here, and an 8-pixel-width band along each ring (voter) will be yielded. By superimposing the *N* voters, the target center X^*^, which is identified by the highest peak, can be located.

(6) $$X^{*}=\underset{x}{\text{argmax}}\sum_{n=1}^{N}W_{n}(x)$$

As the histogram based mean shift tracking is rotational invariant, the voted result given by Equation (6) is also rotational invariant.

This method can be deemed as a geometric constrained clustering method. However, as it does not need any complicated clustering parameters, our voting method is more practical in real-time application.

### 3.3. Scale Space Searching

In the above analysis, we have assumed that the target is in fixed-scale mode. However, in a practical imaging system, the target image will become bigger and bigger, like the aircraft approaching the target. Therefore, how to achieve scale invariance is of great concern in our tracking. Fortunately, as the scale difference can be easily identified by changing the size of the kernel radius, which corresponds to the patch size, the histogram based tracking is very convenient to achieve scale searching.

Suppose that the scale changes between two adjacent frames is within [*s*_0_, *s*_1_], we just need to change the kernel radius from *r*_0_*·s*_0_ to *r*_0_*·s*_1_ with a proper step in the PDF statistics for each frame and to conduct the tracking and voting processes accordingly. Then each searching layer will produce an optimal voting result and a candidate target center. Obviously, the voting results can be used to determine the real target center, which corresponds to the maximal voting. Let *s_i_* be the scale factor in one searching, *s_i_ ∈* [*s*_0_*, s*_1_], and ${\left\{x_{i,n}^{*}\right\}}_{n=1,\ldots ,N}$ be the tracking results in layer *s_i_*, and the new voter in this scale becomes

(7) $$W_{i,n}(x)=\frac{1}{2\pi \sigma^{2}}\;\text{exp}\;\left(\frac{-{\left(D_{i,n}(x)-d_{n}\cdot s_{i}\right)}^{2}}{2\sigma^{2}}\right)$$

where $D_{i,n}={\left\|x-x_{i,n}^{*}\right\|}_{2}$, *d_n_* · *s_i_* is the new distance from the *n*th subtemplate to the target center. It should be noticed that as the target size changes, the prior distance *d_n_* must be updated correspondingly. Meanwhile, the new form of the voting Equation (6) becomes

(8) $$X_{i}^{*}=\underset{x}{\text{argmax}}\sum_{n=1}^{N}W_{i,n}(x)$$

After searching all the *s_i_ ∈* [*s*_0_*, s*_1_], the optimal scale factor can be determined by the optimal voting:

(9) $$\mathrm{opt}=\underset{x}{\text{argmax}}\sum_{n=1}^{N}W_{i,n}(X_{i}^{*})$$

and the final target location is $X_{\mathrm{opt}}^{*}$.

Our voting strategy directly utilizes the geometric relationship among the multiple tracking results to locate the target center, without considering the threshold setting problem encountered by the Bhattacharyya, NCC, EMD, and *L* norm *etc*. similarity-based tracking methods. Moreover, the threshold setting problem may become more difficult in high dimensional searching cases [10].

For tracking the next frame, we just need to update the centers of the subtemplates and the prior distances. The update equations are given by

(10) $$x_{n}=x_{n}^{*},\;\;\;d_{n}^{k}=d_{n}^{k-1}\cdot s_{\mathrm{opt}}$$

where *k* is the frame number.

## 4. Experiments

### 4.1. Test Data Sets

We have applied the proposed method to some synthetic videos and some real-life videos. For lack of space, we just post one example in each category. In the synthetic video, the scenes are rotated and enlarged gradually to simulate the real-time imaging sequences of the terminal guidance system. The scene image is downloaded from Google Earth^®^. The real-life video is captured in the laboratory by moving the camera to the target (an envelop on the wall) closer and closer, with some rotating and motion blur in the approaching. The data sets simulate the 3-second (normally 3 ∼ 5 s) aerial target imaging sequences of the terminal guidance procedure.

We use the synthetic video in our test for two reasons: (1) the real terminal guidance video is hard to get; (2) As the target parameters such as position, rotation and scale are all known in each synthetic process, we can test the tracking precision to some further details.

### 4.2. Tracking Comparisons with Synthetic Videos

In the following tests, we chose the original scale adaptive Mean Shift tracking algorithm (OMS), SOAMST [9] and Xu’s spatial color histogram based Mean Shift (SMS) [14] for comparing with the proposed method. As to the OMS, we set *m* = 26 (feature dimension) for both the OMS and our proposed algorithm. Under the condition of not affecting the precision, the smaller *m* is, the less computational cost will be, thus many researches prefer to set it to 16 for color image tracking. However, we have found that 16 is inadequate for the grey-level sequences. The scale searching space is 0.95 *< s_i_ <* 1.05, with a step of 0.05. Theoretically, the target size would not become smaller in the terminal guidance system, but considering there may be divergence in the scale searching, we extend the searching space to the double sides to improve its robustness. As to the SOAMST, which can be downloaded from http://www.comp.polyu.edu.hk/cslzhang/SOAMST.htm, we use its default settings, where video sequences are RGBs and *m* = 16×3, the increased search size is 5, corrected area parameter is 1. As to the SMS, the 2D histogram was set as *m* = 26 (as an improved mean shift algorithm, *m* = 16 is also good), *n* = 3 (spatial bins).

**Figure 3.**
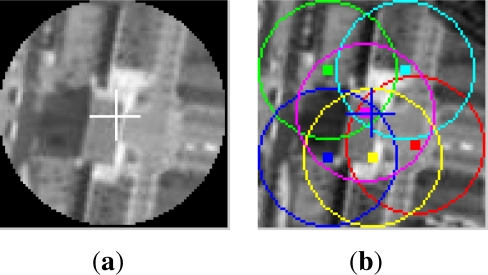
Target templates of the first video. **(a)** template of the OMS, SOAMST and SMS; **(b)** subtemplates distribution of the proposed method.

**Figure 4.**
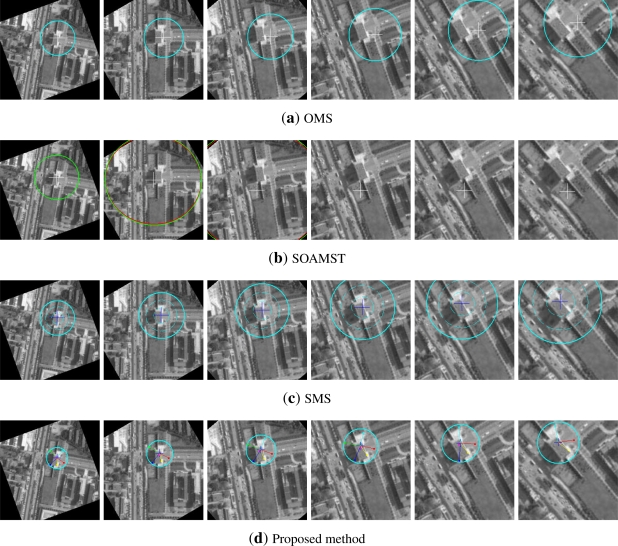
Comparison of the three tracking methods on the first video. **(a)** tracking frames of OMS, the circles denote the real-time kernel radius; **(b)** tracking frames of SOAMST; **(c)** tracking frames of SMS; **(d)** tracking frames of #20, . . ., #70 using our method, where each tracker is marked by one color and their voted target center is marked by “+”, and the circles denote the real-time kernel radius.

Figure 3 is the target templates for the synthetic video; Figure 4 is their corresponding tracking results, respectively. In Figure 3, (a) is the template of OMS, SOAMST and SMS, (b) is the original template image and the selected 6 subtemplates with their centers and region radii marked out. It can be seen that the target template has been cropped to round for achieving the rotational invariance. In Figure 4, (a) is the OMS tracking results, with the target center and kernel radius marked respectively; (b) is the SOAMST tracking results where the outside ellipses indicate the tracked regions of SOAMST; (c) is the SMS tracking tracking results, with the target center and the spatial division lines marked out respectively; (d) is the tracking results for the #20, #30, #40, #50, #60, #70 frames of the proposed method. In each frame, the 6 trackers and their voted target centers are marked with different colors, and the kernel radius is also marked. We can see that in frames #50, #60, #70, as some of the 6 trackers have drifted away, only the valid trackers which give higher vote to the target center are drawn. The original frame size is 512 × 512, and for protruding the target location, we only post the center 256 × 256 subregion of each frame.

It can be seen that: (1) OMS and SOAMST drift away easily; (2) SMS does not drift but its precision is not high (about 4-pixels error); (3) attributed to the multiple subtemplates strategy, the proposed method locks the target center all the time, and its tracking precision achieves pixel level.

### 4.3. Scale Voting

Accurate scale evaluation plays an important role in the tracking. In this test, we are going to discuss the determined scale factors along the tracking by comparing to the ground truth. In OMS and SMS tracking, the scale factors are determined by maximizing the similarities from all the searching layers (the scale factor for the *i*th layer corresponds to *s_i_*); In SOAMST tracking, the scale factor is determined by calculating the square root of the optimal ellipse area proportion. In our method, it is determined by maximizing the voting result.

**Figure 5.**
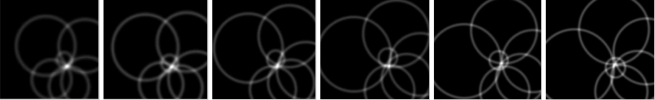
Voting results in the optimal scale layer. Each one corresponds to the frames shown in Figure 4(d).

**Figure 6.**
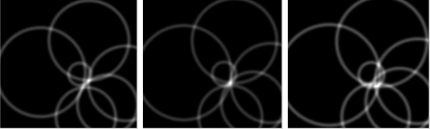
Voting comparison of the three layers in the 54th frame of the first video. From left to right: *s_i_* = 0.95, 1.0, 1.05, and their corresponding voting scores are: 3.30, 4.72, 3.23.

Figure 5 shows the maximum voting of the 3 searching layers (0.95,1.00,1.05). Each frame is chosen by Equation (9), corresponding to Figure 4(d), respectively. In each sub-figure, the band-ring like voters are drawn according to Equation (5), and the brightest pixel indicates the candidate target center in the layer. Taken the 54th frame for example, the voting comparison of the 3 layers is shown in Figure 6, and the corresponding voting scores are 3.30, 4.72, 3.23, respectively. Obviously, the 2nd one is the optimal scale factor, and $s_{\mathrm{opt}}^{54}=1.00$.

**Figure 7.**
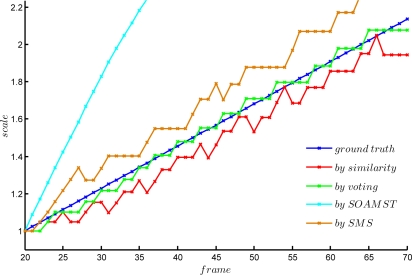
The determined scale factors estimated by the 4 methods comparing to the ground truth.

Figure 7 shows the curves of the cumulated scale variation relative to the first frame (#20). The cumulated relative scale in the *k*th frame can be calculated by

(11) $${\mathrm{scale}}_{k}=\prod_{i=20}^{k}s_{\mathrm{opt}}^{i},k=20,\ldots ,70$$

In the figure, we plot 5 lines: the blue one is the ground truth, which is obtained by the original data in the video synthesizing; the green one is the voted results, the red one is the OMS based results, the brown one is the SMS based results and the cyan one is the SOAMST based results.

It can be seen that: (1) both OMS and the proposed methods can get the true scale factors, however, our method performs better than others; (2) SMS always diverges in some frames and converges rapidly; (3) SOAMST’s result is really bad. Actually, SOAMST is especially good at the cases of target and background with higher contrast, however, there is no salient separation from background and foreground in our applications.

### 4.4. Tracking without Subtemplates Selection

**Figure 8.**
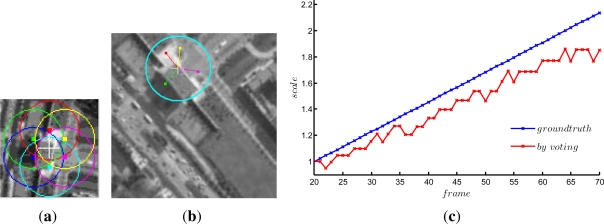
Test results without the subtemplates selection strategy. **(a)** evenly distributed subtemplates; **(b)** tracking results of frame #70; **(c)** determined scale factors comparing to the ground truth.

In order to prove the effectiveness of our subtemplates selection algorithm, we test the proposed method many times by 6 randomly selected subtemplates. For lack of space, we just post one example, as shown in Figure 8, where (a) is the 6 subtemplates whose centers are evenly distributed in a circle with radius of 17 pixels; (b) is the tracking results of the frame #70; (c) is the scale variation curves.

It can be seen that the tracking precision is similar to Figure 4(d), however, the estimated scale factors are not so precise as shown in Figure 7, which may affect some further processes such as estimating the target distance based on the scale factors.

### 4.5. Tracking in Real-Life Scene

**Figure 9.**
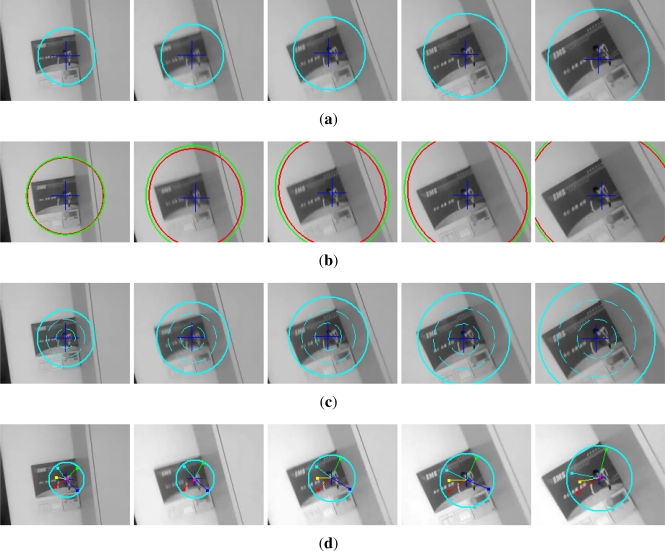
Tracking tests of the 4 methods on the real-life video. **(a)** OMS; **(b)** SOAMST; **(c)** SMS; **(d)** Proposed method.

**Figure 10.**
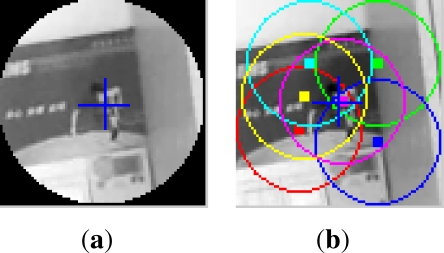
Target templates of the real-life video. **(a)** template of the OMS/SOAMST/SMS; **(b)** subtemplates distribution of the proposed method.

For further testing, we conduct the 4 methods on a real-life video, as seen in Figure 9, where the #50, #60, . . ., #90 frames are posted only. The original frame size is 320 × 240, and for protruding the target location, we only post the center 180 × 140 subregion of each frame. Figure 10 shows the templates of the OMS/SOAMST/SMS (left) and our method (right) in the tests, respectively, with the same size of 80 × 80.

From the tracking frames, we can see that: (1) the OMS still drifts away in the end; (2) SMS does not drift but still has 5 ∼ 10 pixels error; (3) SOAMST and our method locks the target constantly and precisely, and meets the demands of the terminal guidance system. However, as seen in the following computational analysis, our method runs much faster than SOAMST.

From all the tests we can achieve the following conclusions: (1) our subtemplates selection strategy is helpful to improve the tracking robustness; (2) our voting strategy can effectively and accurately determine both the scale factor and the target location; (3) the proposed multiple subtemplates tracking algorithm meets the demand of the terminal guidance system.

### 4.6. Computational Analysis

All the tests are implemented using the MATLAB^®^2011a on a PC with 2.93 GHz CPU (Intel^®^I3-530), 2 GB memory. The initial template size of each tracking is 80 × 80. The computational cost will be increased when the target enlarged gradually. As we see, the OMS/SOAMST/SMS carries on the mean shift iterative processes with a whole template while our method does it with *N*, but much smaller subtemplates, so the computational increase of our method is not high. On average, the OMS and SMS tracks each frame with 200 ms, and our method with 300 ms. As to the SOAMST, it runs very slow as the wrong scale estimation. When tracking on 80 × 80 regions, SOAMST runs more than 1 s for each frame, and that will rise at an exponential rate while the region size increasing.

## 5. Conclusions

Inspired by the multi-part model, this paper presents a multiple subtemplates based mean shift tracking algorithm to achieve a higher tracking precision for the terminal guidance application where the targets are often static such as buildings or slowly moving ships. Relying on proposed rules, an optimized subtemplates selection algorithm is designed. The selected subtemplates maximize the information representation of the original template and minimize their redundancy from each other. A voting strategy to accurately locate the target center by the prior spatial constrain information of those subtemplates is also proposed. Moreover, the voting strategy can easily determine the scale factor and avoid the threshold setting problems which need to be considered in most similarity based methods. Experiments on some videos with static scenes show that the voting strategy can effectively find both the scale factor and the target center, and the proposed method successfully improves the tracking precision of the original mean shift method when rotation and scale changes. Moreover, like other multi-part based methods, the proposed method can also cope with the partly covered target tracking problem.

This paper also provides a solving framework for the histogram based matching or tracking problems, only if the histogram features satisfy rotational invariance, such as the Rotation-Invariant Fast Features (RIFF) [16], which can effectively cope with the differences in light and can be used in the sequences with less information, such as infrared, SAR. Moreover, we can also introduce Xu’s spatial color histogram [14] to our method, which must be able to further improve the tracking precision.
